# Anxiety problems in children and adolescents: a population-based cohort study on incidence and management in Dutch primary care

**DOI:** 10.3399/BJGP.2021.0557

**Published:** 2022-04-20

**Authors:** Lukas BM Koet, Evelien IT de Schepper, Arthur M Bohnen, Patrick JE Bindels, Heike Gerger

**Affiliations:** Department of General Practice, Erasmus Medical Centre, the Netherlands.; Department of General Practice, Erasmus Medical Centre, the Netherlands.; Department of General Practice, Erasmus Medical Centre, the Netherlands.; Department of General Practice, Erasmus Medical Centre, the Netherlands.; Department of General Practice, Erasmus Medical Centre, the Netherlands; Department of General Practice and Family Medicine, University of Bielefeld, Bielefeld, Germany.

**Keywords:** adolescent, anxiety, children, incidence, general practice, treatment

## Abstract

**Background:**

Due to a large strain on youth mental health care, general practice is suggested as an alternative treatment setting for children and adolescents with anxiety problems. However, research on the current management of these children and adolescents within general practice is scarce.

**Aim:**

To investigate the incidence of coded anxiety in general practice using the International Classification of Primary Care (ICPC), and GPs’ management of children and adolescents presenting with anxiety problems.

**Design and setting:**

Population-based cohort study using electronic medical records of 51 212 children (aged 0–17 years) in primary care in the Rotterdam region between 1 January 2012 and 31 December 2018.

**Method:**

Incidence of ICPC codes for anxiety were calculated, then the characteristics of children and adolescents consulting their GP with anxiety and the GPs’ management were assessed qualitatively using quantitative content analysis.

**Results:**

Incidence of ICPC codes for anxiety in children and adolescents was 5.36 (95% confidence interval [CI] = 5.02 to 5.71) per 1000 person–years. Adolescent females had the highest incidence with 14.01 (95% CI = 12.55 to 15.58) per 1000 person–years. Of the 381 children and adolescents consulting their GP with an initial anxiety problem (median age 13.3 years, 40.4% male), GPs referred 59.3% to mental health care in the first year while 26.5% of children and adolescents were managed by a specialised practice nurse within general practice. Of the 381 children and adolescents, 10.5% received psychiatric medication during the first year, with the trend being for increased prescriptions during adolescence.

**Conclusion:**

In general practice children and adolescents frequently received one of two ICPC codes for anxiety, especially adolescent females. Most presenting to their GP with anxiety problems are referred externally or seen by a specialised practice nurse within general practice.

## INTRODUCTION

Anxiety disorders form the most common mental health problem in children and adolescents and cause significant burdens.^1^ A recent meta-analysis estimated the global prevalence of anxiety disorders among children and adolescents at 6.5%.^2^ Anxiety disorders have significant negative effects on quality of life and the overall development of affected children and adolescents and their next of kin,^3^^–^6 and are associated with an increased risk of suffering from mental health disorders in adulthood.^7^^–^10

Despite the existence of effective treatments,^11^^–^13 paediatric anxiety disorders seem under-recognised and undertreated.^14^^–^20 Factors like stigmatisation, financial costs, or limited access to services, for example, waiting lists, form major barriers to appropriate care.^21^ General practice has been advocated as an appropriate treatment setting for paediatric mental health problems because it is easily accessible and not associated with stigmatisation.^22^

GPs already play an important role in the help-seeking process to care, being a familiar and trusted source of help for children, adolescents, and parents.^23^^–^25 In the Netherlands, GPs have a gatekeeper role. GPs’ care is covered by health insurances, which are compulsory for all. During the past years in pilot projects, youth mental health practice nurses (YMHPNs) have been introduced to Dutch general practice aiming to integrate mental health care to general practice. YMHPNs are involved in managing and referring children and adolescents with psychosocial problems.^26^^,^^27^ YMHPNs work independently under the responsibility of GPs, and are allotted more time per consultation.

Several studies on the prevalence of paediatric anxiety disorders have been published,^2^^,^^6^^,^^28^^–^33 but there is sparse knowledge on the incidence of paediatric anxiety symptoms and disorders in general practice.^34^^,^^35^

Little is known about how GPs actually manage these problems. In a 2019 UK survey, 51% of GPs felt confident identifying anxiety disorders in children and adolescents, but only 13% felt confident managing them.^36^ More information on how GPs currently manage anxiety problems in children and adolescents is needed.

The authors, therefore, aimed to conduct two analyses: first, to calculate the incidence of International Classification of Primary Care (ICPC)-coded anxiety (P01 or P74) among children and adolescents. Second, using a broader sample of children and adolescents with anxiety problems, to describe the characteristics of children and adolescents presenting to their GP, and the GPs’ management of these problems using qualitative analyses.

## METHOD

### Design

A population-based retrospective cohort study of children and adolescents (aged 0–17 years) registered in the Rijnmond Primary Care Database (RPCD) between 1 January 2012 and 31 December 2018 was performed. The RPCD is a region-specific derivative of the Integrated Primary Care Information (IPCI) database, focused on the greater Rotterdam area.^37^^,^^38^ The RPCD contains pseudonymised longitudinal medical data of general practice patients, including complete GP notes, diagnostic codes, referrals, laboratory findings, GP prescriptions, and specialists’ letters.^37^ Dutch GPs use the International Classification for Primary Care 1 (ICPC-1) to code symptoms and diagnosis.^39^ The RPCD currently contains data for approximately 300 000 individuals.

**Table:** How this fits in

It has been suggested to treat more minors with anxiety problems within general practice, but little information on the actual treatment of these problems in general practice is available. This study shows that children and adolescents in general practice in the Netherlands frequently receive an ICPC code for anxiety. The majority of children and adolescents consulting their GP with a first anxiety problem are referred to mental health care and a quarter of them are seen within general practice by a specialised practice nurse.

### Incidence of ICPC-coded anxiety (P01 or P74; quantitative analyses)

**Figure 1.**
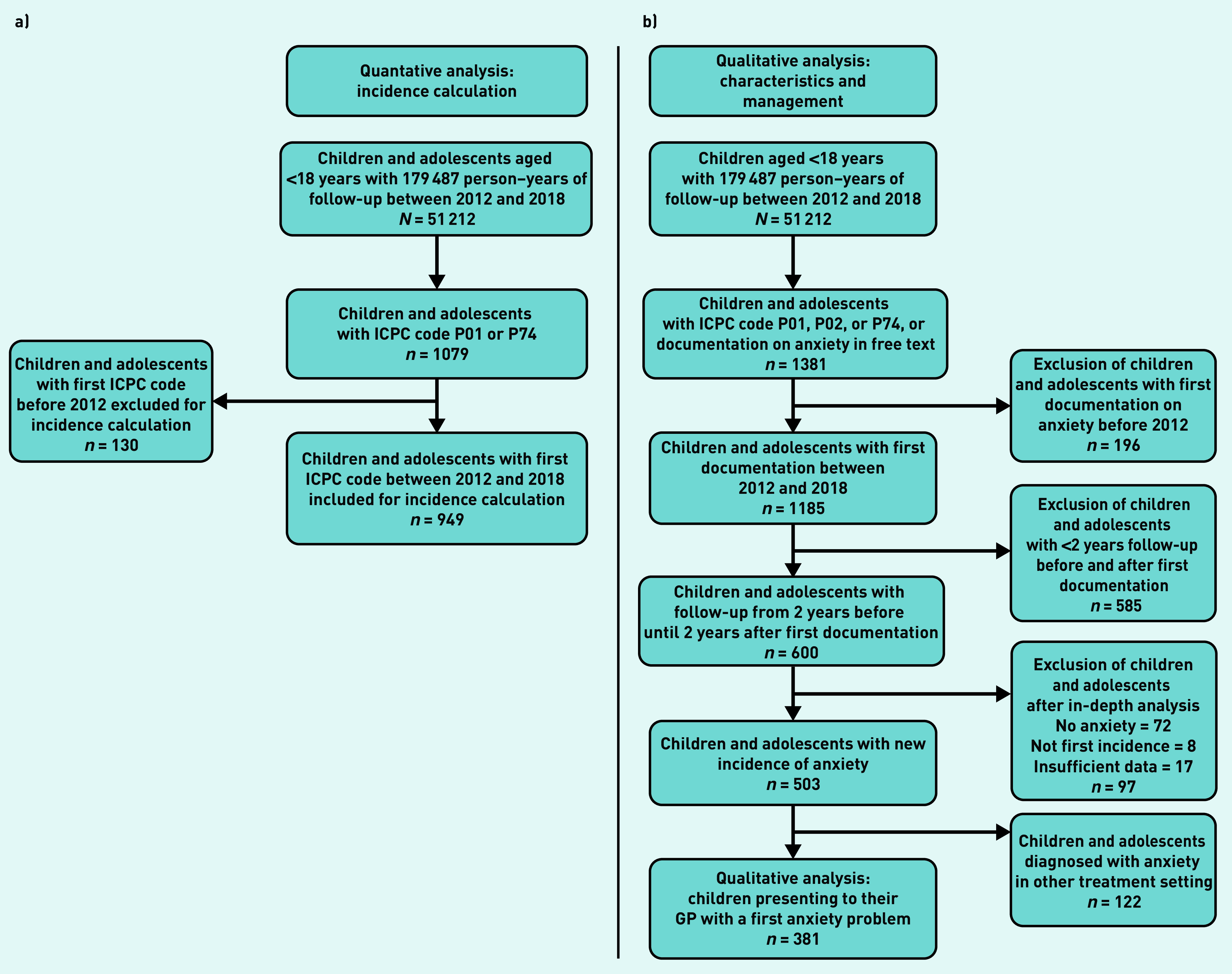
*a) Patient selection for incidence of ICPC-coded anxiety calculation; b) qualitative analyses of children presenting with anxiety problems. ICPC = International Classification of Primary Care.*

The incidence of ICPC-coded anxiety: P01 (feeling anxious/nervous/tense) or P74 (anxiety disorder/panic disorder) was calculated (see flowchart in Figure 1). For pragmatic reasons, the authors combined the codes P01 and P74 for the incidence calculation because the codes were used interchangeably by GPs in the database. The following characteristics of children and adolescents receiving a first ICPC code P01 or P74 were extracted from the database: date of coding, age, and sex.

### Case selection (qualitative analyses)

Since paediatric anxiety problems in general practice often reflect symptoms rather than strict diagnostic categories, the authors were interested in children and adolescents presenting with anxiety problems to their GP. Therefore, selected cases do not necessarily fulfil the diagnostic criteria of anxiety disorders but are rather a broader range of anxiety problems, for example, anxiety problems of short duration.^40^ In this study, children and adolescents presenting with a first anxiety problem to their GP (Figure 1 and Supplementary Table S1) were selected. A search algorithm combining ICPC codes P01 and P74, ICPC code P02 (acute stress reaction/post-traumatic stress syndrome), and a free-text search for the terms ‘anxiety disorder’ and ‘anxiety problems’ were used to increase the sensitivity for detecting children and adolescents with anxiety problems. To make valid inferences on the run-up period and GPs’ management, the authors limited inclusion to children and adolescents with valid database information from 2 years before until 2 years after the first record of their anxiety problem. Full medical files from this period (600 cases) were examined by the first author (principal investigator) to exclude cases not registering an initial anxiety problem (see Supplementary Table S1). Unclear cases were reviewed by the second author and final decisions regarding their addition were consensual (first and second authors). The ICPC code P02 had a low positive predictive value (PPV) for anxiety problems and was therefore not included in the quantitative analysis (see Supplementary Table S2).

### Characteristics of children and adolescents presenting with anxiety problems and GPs’ management (qualitative analyses)

The following variables were extracted automatically for each case: age at presentation, sex, history of psychosocial problems (any P code/any Z code) and healthcare use (any blood tests and number of consultations with GP) in the 2 years before presentation, pharmacological management from 2 years before until 2 years after presentation (psychiatric medication including antipsychotics, antidepressants, anxiolytics, and hypnotics [Anatomical Therapeutic Chemical {ATC} classification system N05–N07] and beta-blockers [ATC C07]).

The following information was extracted manually by the first author using quantitative content analysis, by reading medical files from 2 years before until 2 years after presentation, and by counting the respective occurrence: healthcare use in the 2 years before presentation, that is, any referrals to specialist medical care and any visits to emergency care; associated factors described in the GPs’ notes/specialists’ letters in 2 years before until 2 years after presentation, that is, marital status of parents, presence of domestic violence/maltreatment, victim of sexual violence/crime/bullying, fear of failure, sleep problems, school problems/absenteeism, and concentration problems; and GPs’ management in the first year after presentation, that is, any referral, type of referral, and number of consultations until referral.

Referrals were classified as: a) referral to primary or specialised mental health care; and b) referral to paediatrician.

Additionally, involvement of YMHPNs within general practice and the number of consultations with YMHPNs were extracted manually.

### Statistical analysis

Incidence rates were determined by dividing the number of cases that received a first ICPC code for anxiety (P01 or P74) by the total number of person–years-at-risk (PYAR) and are expressed per 1000 person–years. Incidence rates were analysed by age group (young children: aged 0–6 years; children: aged 7–12 years; and adolescents: aged 13–17 years) and sex (male versus female).^41^ PYAR was defined as actual time at risk in years that children and adolescents (aged <18 years) without ICPC-coded anxiety contributed to the study’s database.

Descriptive statistics were used to describe patient characteristics and the GPs’ management. Statistical analysis of differences in proportions between sex and age categories were performed using the Pearson *χ*^2^ test and Fisher’s exact test.

Student’s *t*-test was used for testing statistical significance of observed means. Due to the exploratory nature of this study, no adjustments for multiple testing were performed. Analyses were performed using R (version 4.0.0).

### Reporting and ethical considerations

The authors adhered to the RECORD guidelines for the reporting of studies using electronic health records.^42^

## RESULTS

### Study population

In total, 51 212 children and adolescents aged 0–17 years were included in the cohort between 2012 and 2018. The median age was 8.7 years and 50.8% were male. The number of registered children and adolescents in the database increased from 21 140 children and adolescents in 2012 to 35 190 children and adolescents in 2018. The general characteristics of children and adolescents remained comparable over time (see Supplementary Table S3).

### Incidence of ICPC-coded anxiety (P01 or P74; quantitative analyses)

**Figure 2.**
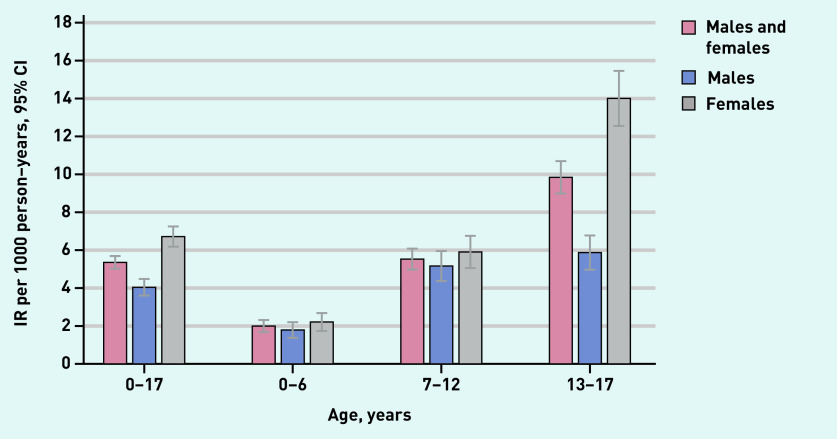
*Incidence of ICPC-coded anxiety per age category.* *ICPC = International Classification of Primary Care.* *IR = incidence rate.*

During the study period, 949 children and adolescents were coded for the first time with ICPC code P01 or P74. Median age at coding was 13.2 years (interquartile range [IQR] 9.1–16.3) and 61.6% were female (data not shown). The overall incidence was 5.36 (95% confidence interval [CI] = 5.02 to 5.71) per 1000 PYAR. The incidence was higher for females than males with an incidence rate ratio (IRR) of 1.66 (95% CI = 1.46 to 1.89). Females aged 13–17 years had the highest incidence rate with 14.01 (95% CI = 12.55 to 15.58) per 1000 PYAR (see Figure 2 and Supplementary Table S4 and S5 for incidence rate per category and year).

### Characteristics of children and adolescents presenting with anxiety problems (qualitative analysis)

In total, 381 children and adolescents presenting with anxiety problems to their GP (Figure 1) were included in the study. Of these, 154 were male (40.4%) and 227 were female (59.6%) (Table 1). Median age at presentation was 13.3 years (data not shown). Females were older at presentation than males (14.0 years versus 12.2 years, *P* = 0.02).

**Table 1.** Comorbidities and healthcare use in 2 years before presentation with an anxiety problem

**Comorbidities and healthcare use**	**Overall *n* = 381**	**Males *n* = 154**	**Females *n* = 227**	**Aged 0–6 years *n* = 37**	**Aged 7–12 years *n* = 146**	**Aged 13–17 years *n* = 198**	**Male versus female *P*-value^a^**	**Age categories *P*-value^a^**
Median consultations in general practice (IQR)	5 (3–9)	5 (3–8)	5 (3–9)	7 (5–9)	4 (2–7)	5 (3–9)	0.16	0.002
Previous mental health problem (ICPC, any P code), %^b^	22.6	21.4	23.3	21.6	28.1	18.7	0.75	0.12
Previous social problem (ICPC, any Z code), %	9.4	12.3	7.5	18.9	8.2	8.6	0.16	0.12
≥1 prescription of psychiatric medication, %	6.0	5.2	6.6	0.0	3.4	9.1	0.73	0.03
≥1 blood test, %	35.4	31.8	37.9	21.6	24.7	46.0	0.27	<0.001
≥1 referrals to mental health or hospital specialist, %	42.3	46.8	39.2	62.2	47.3	34.8	0.17	0.003
≥1 visit to emergency department in 1 year before diagnosis, %	24.9	29.9	21.6	48.6	24.0	21.2	0.09	0.002

a *Percentages among males compared with females, percentages among age categories compared with the χ^2^ test. These hypothesis tests are of exploratory nature, and are therefore not corrected for multiple testing.*

b *Any P code other than P01, P02, or P74. ICPC = International Classification for Primary Care. IQR = interquartile range.*

During the 2-year run-up period, children and adolescents consulted their GP a median of 5 times in 2 years (Table 1). In this period, 22.6% were coded with a psychosocial problem (any P code other than P01, P02, or P74), and 6.0% received ≥1 prescriptions of psychiatric medication (ATC N05–N07). Healthcare use in the run-up period was comparable for males and females. There was a trend in different healthcare use between age groups, with young children (aged 0–6 years) being referred more often and adolescents (aged 13–17 years) undergoing more blood tests. The medical dossiers frequently contained information on the presence of problems associated with anxiety (see Supplementary Table S6).

### GPs’ management (qualitative analysis)

During the first year after presentation with an anxiety problem, children and adolescents had a median of 1 consultation (IQR 1–2) with their GPs concerning the anxiety problem (Table 2). In total, 72.7% of children and adolescents were referred within the first year, either externally to mental healthcare providers or paediatricians, or internally to the YMHPN. If additional mental health providers were engaged, in 78.7% of cases the GP decided to involve them at the first consultation (data not shown). In 59.3% of cases children and adolescents were referred to external mental healthcare providers. In cases of referral to paediatricians (5.0%), anxiety problems were accompanied by physical symptoms. Overall, 26.5% of cases were seen by a YMHPN, with a median of 3 contacts (IQR 1–4.5). Of the 57.4% of children and adolescents seen by a YMHPN, 15.2% were also referred externally to mental healthcare providers in the year after presentation (Table 2). Usually, these children and adolescents were first seen by a YMHPN and later referred to mental healthcare providers but the exact order of referral could not always be verified.

**Table 2.** GPs’ management of anxiety problems in first year after presentation

**Management**	**Overall *n* = 381**	**Males *n* = 154**	**Females *n* = 227**	**Aged 0–6 years *n* = 37**	**Aged 7–12 years *n* = 146**	**Aged 13–17 years *n* = 198**	**Male versus female *P*-value^a^**	**Age categories *P*-value^a^**
Median contacts with GP for anxiety (IQR)	1 (1–2)	1 (1–2)	1 (1–2)	1 (1–2)	1 (1–2)	0.87	0.87	1 (1–2)
Referral for anxiety either external or by internal involvement of YMHPN, %	72.7	67.5	76.2	86.5	78.1	66.2	0.08	0.007
External referral to mental health care, %	59.3	58.4	60.0	78.4	71.9	46.5	0.86	<0.001
External referral to paediatrician, %	5.0	4.5	5.3	5.4	4.8	5.1	0.09	0.13
Involvement of YMHPN, %	26.5	20.1	30.8	8.1	21.2	33.9	0.03	<0.001
Median contacts with practice nurse (IQR)	3 (1–5)	2 (1–5)	3 (1–4)	2 (2–2)	2 (1–4)	3 (1–5)	0.52	0.40
Involvement of YMHPN and external referral mental health care, %^b^	15.2	11.7	17.6	5.4	15.1	17.2	0.15	0.19

a *Percentages among males compared to females, percentages among age categories compared with χ^2^ test. These hypothesis tests are of exploratory nature, and are not corrected for multiple testing.*

b *Children and adolescents seen by the YMHPN and externally referred to mental health care in first year after presentation; 57.4% of children seen by the YMHPN were also externally referred. YMHPN = youth mental health practice nurse.*

Of all children and adolescents referred to mental health care, 40.0% of the medical records contained a specialist letter with conclusive information about diagnosis or treatment. Regarding these children and adolescents, 31.5% received an anxiety disorder diagnosis by a psychologist or psychiatrist, 28.7% received treatment for anxiety problems without receiving a formal anxiety disorder diagnosis, 9.3% were diagnosed with post-traumatic stress disorder, and 30.5% of children and adolescents received other diagnoses (either autism, ADHD, or behavioural disorders) (data not shown).

### Pharmacological treatment

In the first year after presentation, 10.5% of children and adolescents received at least one GP-prescribed psychiatric medication (ATC N05–N07; see Table 3). The prescription rate was the highest for adolescents aged 13–17 years (14.1%).

**Table 3.** Psychiatric medication prescriptions

**Prescription type, %**	**Overall *n* = 381**	**Males *n* = 154**	**Females *n* = 227**	**Aged 0–6 years *n* = 37**	**Aged 7–12 years *n* = 146**	**Aged 13–17 years *n* = 198**	**Male versus female *P*-value^a^**	**Age categories *P*-value^a^**
Psychiatric: first year after diagnosis^b^	10.5	8.4	11.9	2.7	7.5	14.1	0.36	0.03
Psychiatric: second year after diagnosis	8.4	7.1	9.3	2.7	4.1	12.6	0.59	0.01
Psychiatric: 0–2 years after diagnosis	13.6	9.7	16.3	5.4	8.2	19.2	0.09	0.004
SSRI prescription: first year after diagnosis	1.0	0.0	1.8	0.0	0.0	2.0	0.15	0.15
SSRI: second year after diagnosis	1.6	0.6	2.2	0.0	0.0	3.0	0.43	0.06
SSRI: 0–2 years after diagnosis	1.8	0.6	2.6	0.0	0.0	3.5	0.26	0.05
Benzodiazepine: first year after diagnosis	5.5	3.9	6.6	0.0	2.1	9.1	0.36	0.006
Benzodiazepine: second year after diagnosis	2.1	2.6	1.8	0.0	0.0	4.0	0.72	0.03
Benzodiazepine: 0–2 years after diagnosis	6.0	5.2	6.6	0.0	2.1	10.1	0.73	0.004
Beta-blocker: first year after diagnosis	8.7	6.5	10.1	0.0	0.0	16.7	0.29	<0.001
Beta-blocker prescription: second year after diagnosis	2.1	2.0	2.2	0.0	0.0	4.0	1.00	0.04
Beta-blocker prescription: 0–2 years after diagnosis	9.2	6.5	11.0	0.0	0.0	17.7	0.19	<0.001

a *Percentages among males compared to females, percentages among age categories compared with χ^2^ test. These hypothesis tests are of exploratory nature, and are not corrected for multiple testing.*

b ≥*1 psychiatric prescriptions of ATC classification N05–N07. ATC = Anatomical Therapeutic Chemical. SSRI = selective serotonin reuptake inhibitor.*

Benzodiazepines were prescribed to 5.5% of children and adolescents in the first year after presentation, mainly to adolescents, with 9.1% of adolescents receiving at least one prescription. Beta-blockers were only prescribed to adolescents, with 16.7% of adolescents receiving at least one prescription within 1 year.

## DISCUSSION

### Summary

The incidence of ICPC codes P01 and P74 was 5.36 per 1000 PYAR. Females were more often affected than males. The highest incidence rate of 14.01 per 1000 PYAR was in adolescent females. GPs referred 59.3% of children and adolescents presenting with an initial anxiety problem to external mental healthcare providers, YMHPNs were involved in 26.5% of cases. In the year after presentation, 10.5% of children and adolescents, especially adolescents, received a psychiatric prescription.

In general practice, children and adolescents frequently receive one of two ICPC codes for anxiety, especially adolescent females. Most children and adolescents presenting to their GP with anxiety problems are referred externally or seen by a specialised practice nurse within general practice.

### Strengths and limitations

This study combines the advantages of a large dynamic cohort (using quantitative data from medical files) with in-depth qualitative analyses of selected medical files. In the qualitative analysis, the authors reviewed children and adolescents with a clinically relevant follow-up period of ≥4 years who presented to their GP with anxiety problems. The authors were able to create a sensitive search algorithm for children and adolescents presenting with first anxiety problems to their GP using both ICPC codes and free text (see Supplementary Table S4 and S5).

This study has limitations. First, for logistical reasons, GPs’ notes are often concise, and information on symptoms and associated problems might not always have been documented. Second, incorrect or imprecise coding by healthcare professionals is a disadvantage of using electronic healthcare databases and can cause under- and overestimation of symptom and disease frequency.

In the presented qualitative search, the authors found that children and adolescents coded with P02 sometimes had relevant anxiety complaints without receiving an ICPC code for anxiety (P01 or P74). Therefore, the incidence rate of ICPC coded anxiety (P01 or P74) may underestimate the frequency of paediatric anxiety problems.

Third, because GPs do not usually give a formal diagnosis of anxiety disorders nor do they use standardised screening/classification tools, it is difficult to differentiate between milder and severe anxiety problems in the database used. Fourth, only GPs’ prescriptions of medications can be extracted from the RPCD, without clarity on whether the medication was started by the GP or a specialist.

Fifth, the RPCD consists of practices in a restricted (sub)urban area, so it remains uncertain whether the present results are generalisable to other regions.

Finally, not all practices in the RPCD had a YMHPN during the study period. Because of anonymised information, the authors were not able to differentiate between practices with and without YMHPNs in the analyses.

### Comparison with existing literature

In a UK primary care registry study and a Danish secondary care registry study the incidence of anxiety disorders varied between 1.8 and 2.6 cases per 1000 PYAR,^34^^,^^35^ considerably smaller incidences as compared with the presented study’s incidence of ICPC-coded anxiety of 5.36. In the present study females had an increased risk of receiving an ICPC code for anxiety compared with males (IRR 1.66 [95% CI = 1.46 to 1.89]), which is in line with previous studies.^28^^,^^34^^,^^35^ Compared with the present findings, a Norwegian study using primary and secondary healthcare data found a higher prescription rate of anxiolytic medication.^43^ Further, the present finding that GPs frequently involve additional mental health specialists and YMHPNs when confronted with children and adolescents with a new anxiety problem confirms conclusions from previous studies that GPs refrain from managing children and adolescents with psychological problems,^18^^,^^36^ and that GPs saw their role mainly as gatekeepers, referring children and adolescents to specialised healthcare providers.^18^

Differences between the present findings and findings from other countries are probably partially explained by methodological differences, for example, differences in inclusion criteria, but may also indicate relevant differences in the occurrence and management of anxiety problems between countries.

### Implications for research and practice

In line with other studies, the authors found a peak incidence of ICPC-coded anxiety in adolescent females.^28^^,^^34^^,^^35^ Therefore, it seems relevant for GPs to consider anxiety problems in children and adolescents, especially adolescent females, presenting with possibly related problems, such as headaches, recurrent abdominal pain, and sleep problems.^44^^–^46 The hesitancy of GPs to manage paediatric anxiety problems combined with a comparably high prescription rate of benzodiazepines and beta-blockers raises concerns regarding the adequate management of paediatric anxiety problems in general practice, given both medications are not routinely indicated for children and adolescents, and that benzodiazepines pose a risk of abuse.^47^ In this context, it seems advisable for GPs to refer not only younger children but also adolescents with anxiety problems to additional screening and counselling by a mental healthcare professional, rather than prescribing benzodiazepines or beta-blockers.

Since 2015, YMHPNs have been introduced to Dutch general practices in order to support the management of psychological problems within general practice settings.^26^^,^^27^ The present study shows that YMHPNs were already involved in the management of more than one-quarter of children and adolescents aged 7–17 years with anxiety problems. Future research should investigate whether the introduction of YMHPNs has improved the availability of treatment opportunities for children and adolescents with anxiety problems and should evaluate the effectiveness of the shared approach involving GPs and YMHPNs. If proven effective, the introduction of YMHPNs could offer an integrational solution to the observed treatment gaps for paediatric psychological problems, and to the apparent hesitancy of GPs to become involved with psychological problems.
